# Alginate Microbeads for Trapping Phenolic Antioxidants in Rosemary (*Rosmarinus officinalis* L.): Multivariate Optimization Based on Bioactive Properties and Morphological Measurements

**DOI:** 10.3390/gels11030172

**Published:** 2025-02-27

**Authors:** Gizem Toprakçı, İrem Toprakçı, Selin Şahin

**Affiliations:** Chemical Engineering Department, Faculty of Engineering, Istanbul University-Cerrahpasa, Avcilar, 34320 Istanbul, Türkiye; gizem.toprakci@bshg.com (G.T.); irem.toprakciyuksel@iuc.edu.tr (İ.T.)

**Keywords:** Biomaterials, hydrogel, encapsulation, ionic gelation, antioxidant

## Abstract

Medical and aromatic plant extracts are often very sensitive to environmental, gastrointestinal, and processing conditions despite their health benefits. Therefore, they can be rapidly inactivated. Microencapsulation is used to overcome such challenges. In this study, phenolic antioxidants from rosemary (*Rosmarinus officinalis* L.) were encapsulated in alginate beads by means of ionic gelation. A Box–Behnken design with response surface methodology (BBD–RSM) was used with three numeric factors (calcium chloride concentration, alginate concentration, and hardening time) to achieve the best formulation in terms of encapsulation efficiency, antioxidant activity, and morphological characteristics. Generally, the sodium alginate concentration of the microbeads was the most critical factor (*p* < 0.0001) for the quality of the products. The optimal encapsulation conditions were accessed using concentrations with almost 6% calcium chloride and 2% alginate, and a time of 10 min for bead hardening in order to obtain the highest responses (30.01% encapsulation efficiency, 7.55 mg-TEAC/g-DM of antioxidant activity value as measured by the DPPH method, a sphericity factor of 0.05, and a roundness of 0.78). At the optimum point, the microbeads were determined to be spherical in shape, and the bulk density value was measured as 0.34 ± 0.01 g/mL.

## 1. Introduction

Microencapsulation is a rapidly developing technology that is applied in specific fields, such as the pharmaceutical and food industries, since the microencapsulation technique can impart specific useful properties to natural ingredients. There are various benefits of using this technology, such as protecting the active material to be coated against external factors (humidity, temperature, air, and light), preventing the loss of taste and odor substances during storage, better preservation of physical properties, and facilitating the transportation of the substance by coating it, ensuring that it works in the right place and at the right time [1]. Moreover, oxidative deterioration can be overcome by preventing undesirable interactions between the materials to be coated [2]. Additionally, the controlled release of aroma substances into the environment can be provided thanks to the technology of covering the flavor with biofilm [3]. This kind of physical barrier around probiotic microorganisms can also protect against adverse environmental conditions in order to maintain the viability of microorganisms [4].

Microencapsulation is defined as a physical entrapment method based on the capture of liquid or gas droplets of small solid particles with the help of thin film layers or polymer capsules made of proteins, carbohydrates, and lipids [5]. In a broad sense, it is the name given to the coating of a substance with a uniform film in the form of very small particles. Ionic gelation is an interesting physical-chemical method for the microencapsulation of hydrophilic active substances [6]. It is feasible due to its simplicity without complex equipment [7]. Additionally, it is a highly cost-effective system in terms of economy since it operates at low temperatures and does not require organic solvents [8]. In this case, it is an excellent alternative for heat-sensitive bioactive systems. It is also a rapid system. If the optimal conditions for the related microencapsulation system are achieved, high encapsulation efficiency values (>80%) are attained [9]. Concerning the shell material, sodium alginate (generally known as alginate) is mostly used for the microencapsulation of bioactive materials due to this natural biopolymer’s advantages, such as biodegradability, non-toxicity, cost efficiency, and easy gel formation [10,11]. Natural products such as astaxanthin from the microalga *Haematococcus pluvialis* [12], betacyanins from the peel of red dragon fruit [13], anthocyanins from haskap berries [14], *Gardenia* yellow pigment from its fruit [15], phenolic antioxidants from lemon balm [11], lycopene from watermelon [16], phenolic antioxidants from papaya leaf [17], gallic acid [18], apigenin [19], and stevia crude extract [20] have been encapsulated in alginate beads. On the other hand, the effect of the polymer matrix on the bioavailability of phenolic compounds is an important issue. The encapsulation process typically involves the ionic gelation of sodium alginate in the presence of divalent cations (calcium ions), causing the formation of calcium alginate beads. This gelation process effectively traps bioactive substances within the beads, protecting them from environmental factors and controlling their release [21,22].

In this study, phenolic antioxidants from rosemary (*Rosmarinus officinalis* L.) were used as active materials since their biological activities (anticancer, antibacterial, antifungal, antioxidant, hepatoprotective, anti-inflammatory, and anti-hyperglycemic properties) were reported by various in vitro and in vivo studies [23,24,25,26,27]. Studies on the encapsulation of rosemary extract are scarce. Visentin et al. used the supercritical antisolvent process for this purpose [28]. Rashidaie Abandansarie et al. encapsulated the hydrophilic rosemary extract in basil seed gum and soybean protein via freeze-drying [29]. Kanakidi et al. used the spray-drying method to encapsulate the hydrophilic extract in maltodextrin combined with gum arabic [30]. Recently, Nutrizio et al. applied high-voltage electrical discharge in calcium alginate/zein/hydroxypropyl methylcellulose to encapsulate the hydrophilic rosemary extract [31]. There are only two reports on alginate-based encapsulation for rosemary extracts. In the first study, six species of aromatic herbs, including rosemary, were compared under the same ionic gelation conditions [32], while the second study compared two aromatic plant extracts, including rosemary, in alginate beads to extend the shelf-life of yogurt [33]. In the current study, unlike the previously published studies, the ionic gelation method has been developed to obtain the best encapsulation efficiency, antioxidant activity, and morphological measurements. For this purpose, a Box–Behnken design with response surface methodology (BBD–RSM) was used with three numeric factors (calcium chloride concentration, alginate concentration, and hardening time for the alginate beads) and three levels. BBD–RSM was specifically selected since it has fewer runs than three-level factorials. Morphological measurements were used to achieve the perfect spherical shape of the beads through the sphericity factor (SF) and roundness (Rn) values, while the antioxidant activity of the products was determined using the in vitro DPPH (2,2-diphenyl-1-picryl-hydrazyl-hydrate) free radical scavenging activity assay. The main purpose of using BBD–RSM in this study is to determine the best formulation for the encapsulation of rosemary phenolics, to evaluate the effects of the factors affecting the production process, and to improve the biological and physical properties of the obtained products by defining the optimum conditions.

## 2. Results and Discussion

### 2.1. Optimization of the Encapsulation Process Based on BBD–RSM

**Figure 1:**
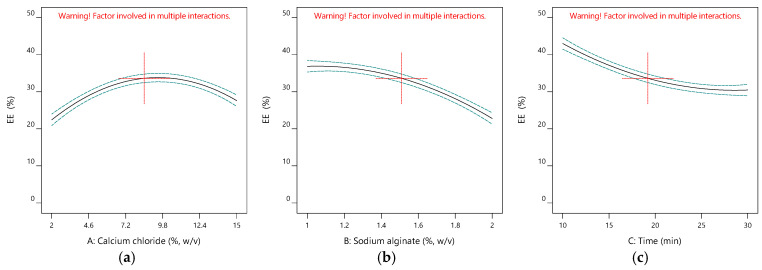
Influence of calcium chloride (**a**), sodium alginate (**b**), and time (**c**) on encapsulation efficiency.

**Figure 2:**
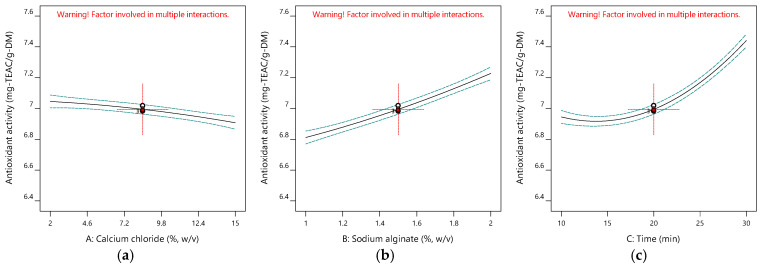
Influence of calcium chloride (**a**), sodium alginate (**b**), and time (**c**) on antioxidant activity.

**Figure 3:**
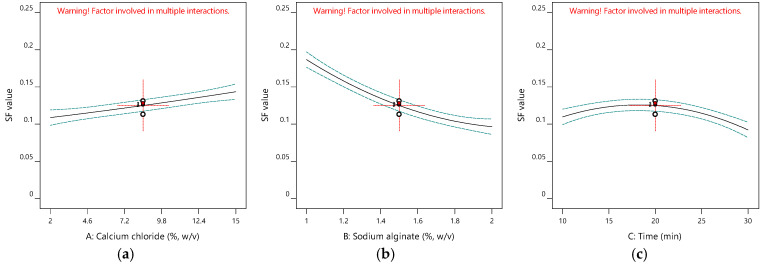
Influence of calcium chloride (**a**), sodium alginate, (**b**) and time (**c**) on the sphericity factor.

**Figure 4:**
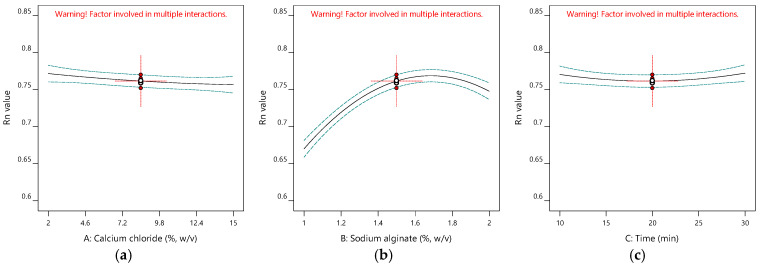
Influence of calcium chloride (**a**), sodium alginate, (**b**) and time (**c**) on roundness.

**Figure 5:**
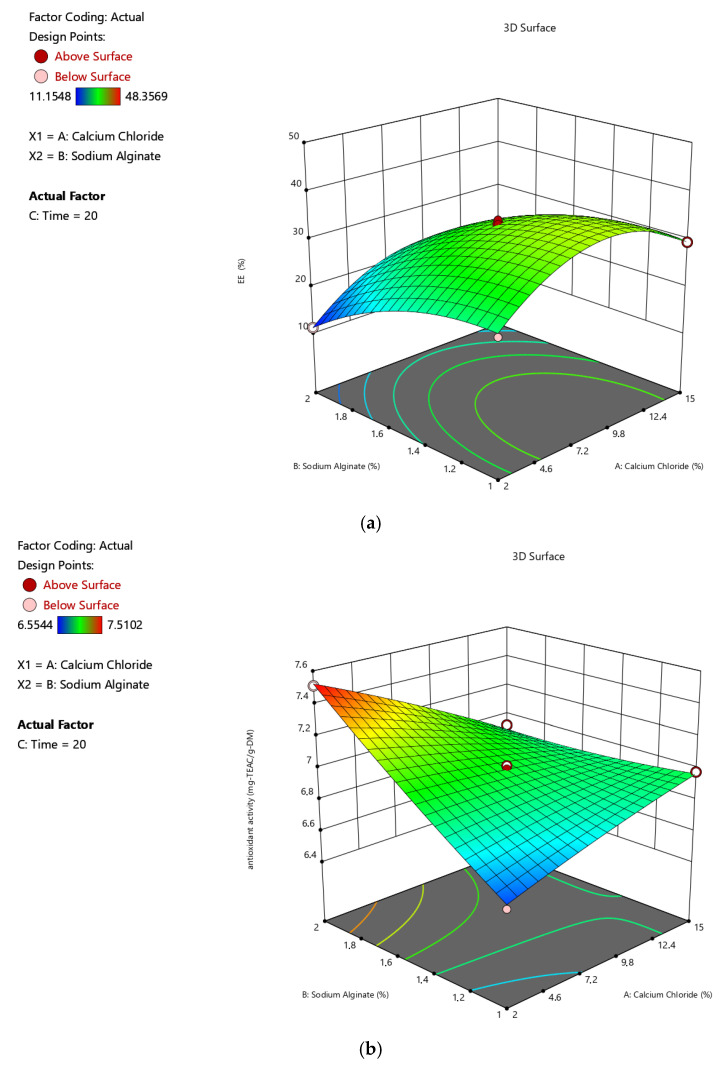

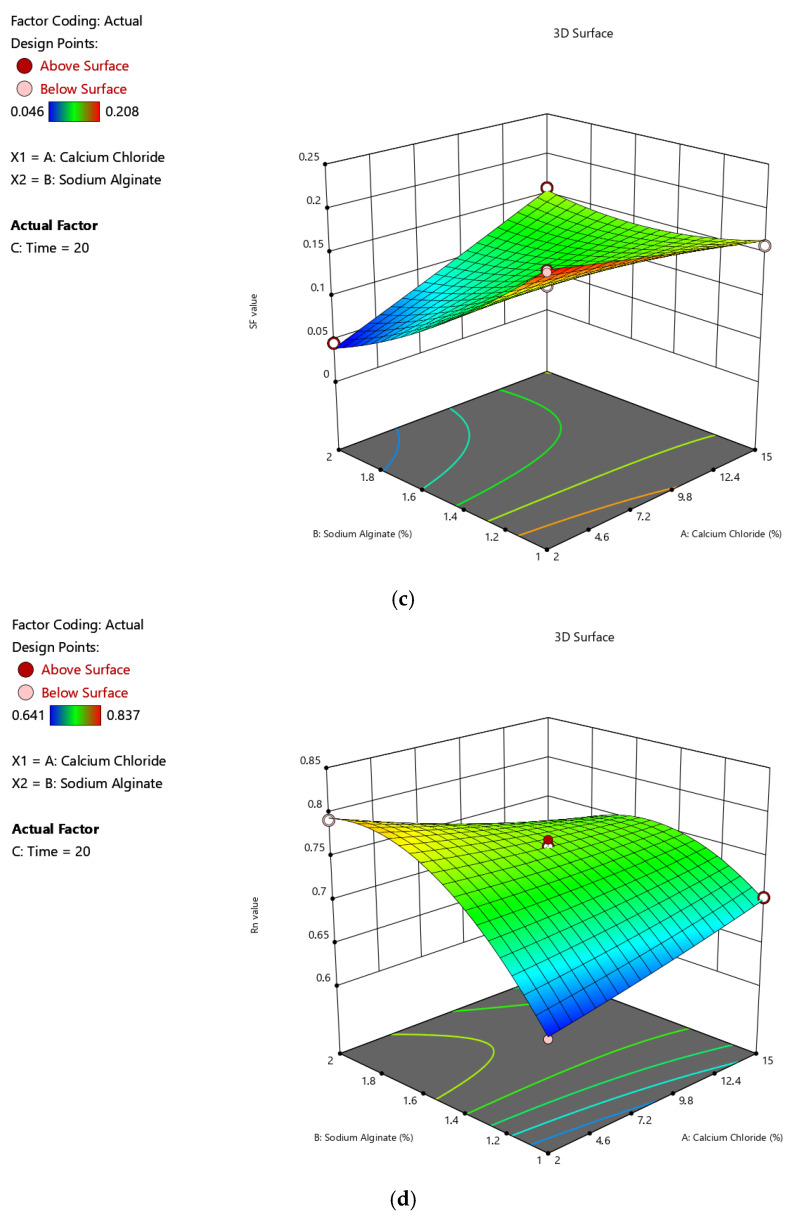
Effects of calcium chloride concentration and sodium alginate concentration on the (**a**) encapsulation efficiency, (**b**) antioxidant activity, (**c**) sphericity factor (SF), and (**d**) roundness (Rn) values of the microcapsules, including the phenolic antioxidant-rich extract from rosemary (*Rosmarinus officinalis* L.).

**Figure 6:**
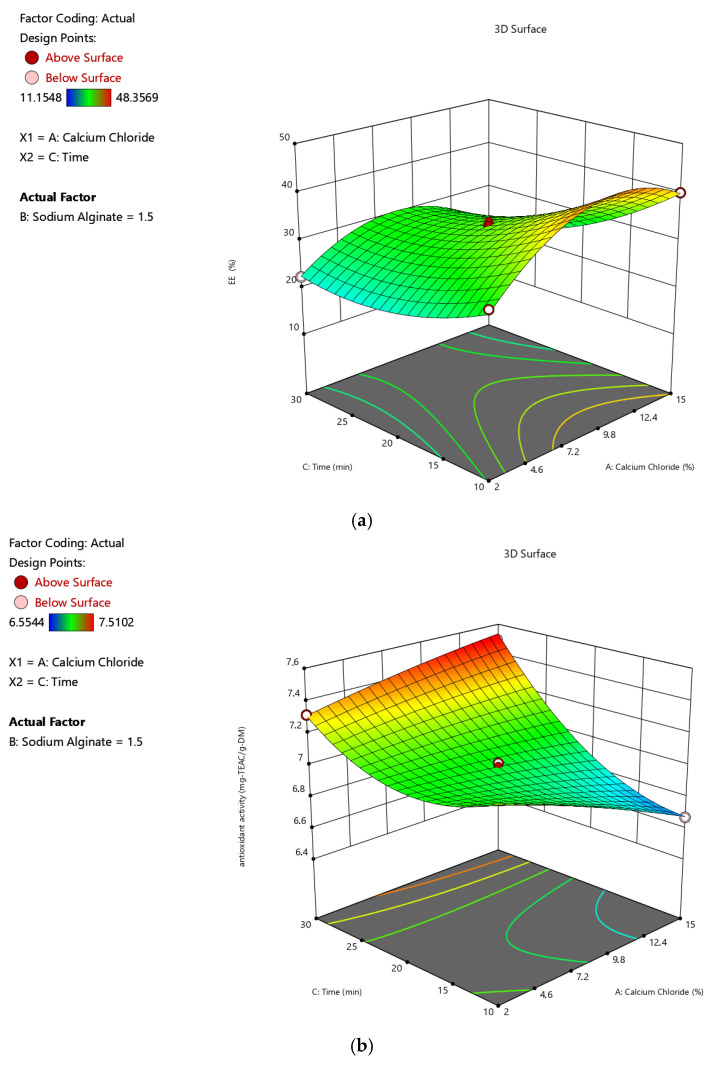

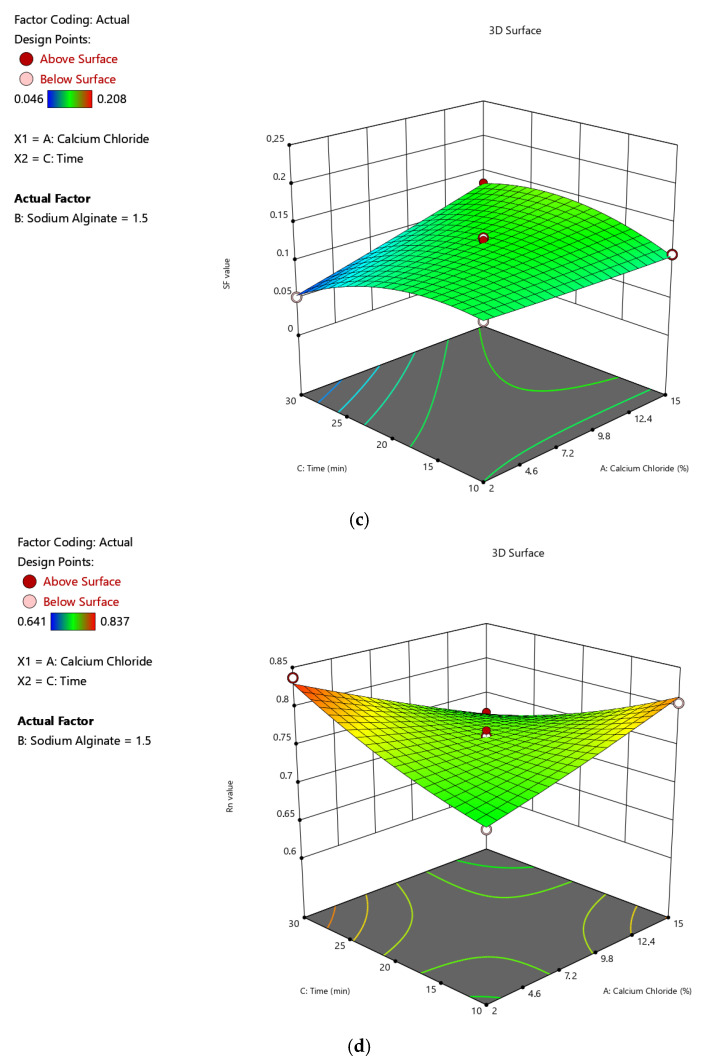
Effects of the calcium chloride concentration and hardening time on the (**a**) encapsulation efficiency, (**b**) antioxidant activity, (**c**) sphericity factor (SF), and (**d**) roundness (Rn) values of the microcapsules, including the phenolic antioxidant-rich extract from rosemary (*Rosmarinus officinalis* L.).

**Figure 7:**
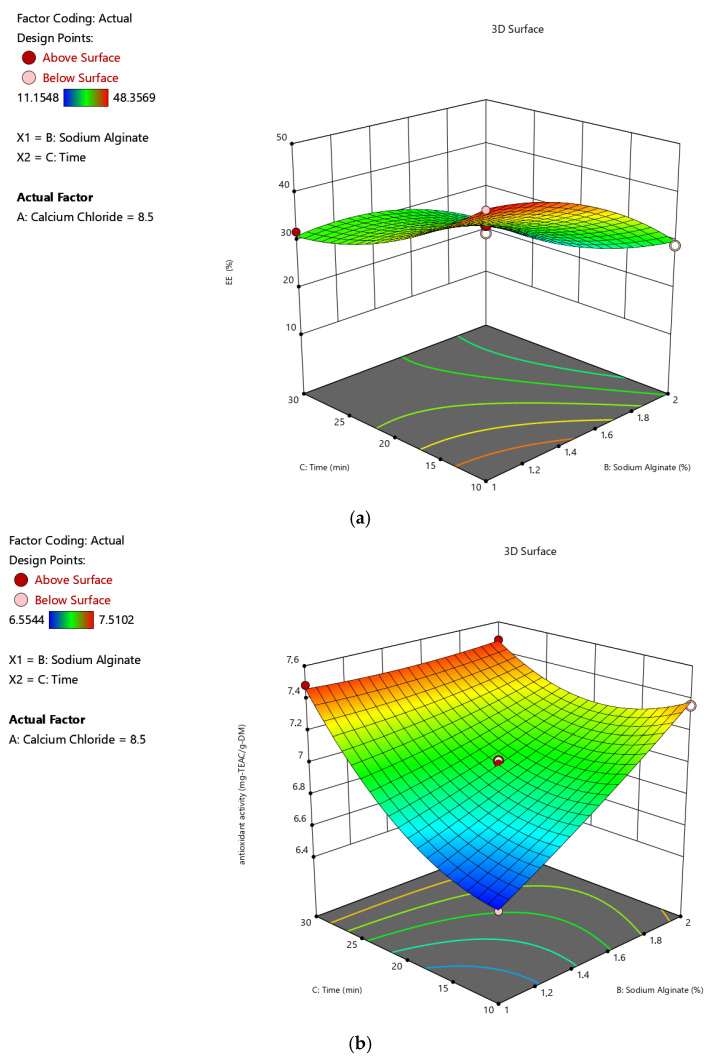

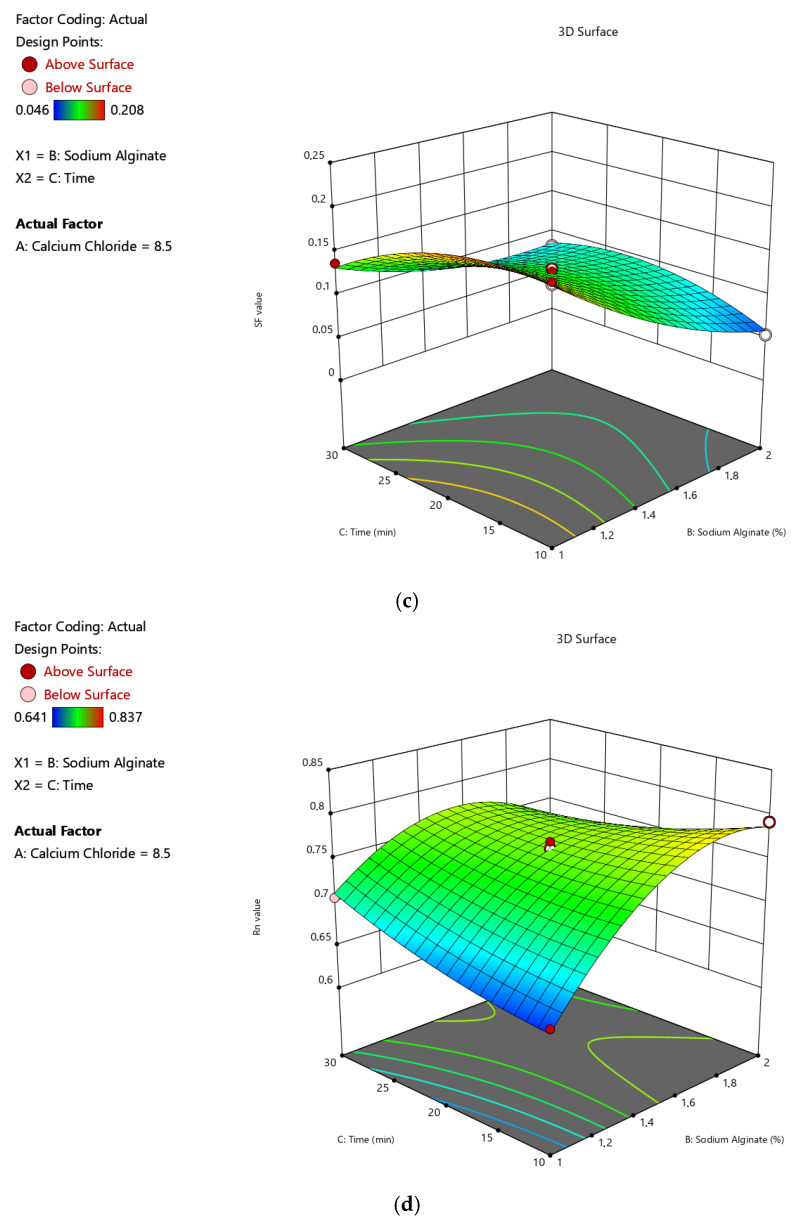
Effects of the sodium alginate concentration and hardening time on the (**a**) encapsulation efficiency, (**b**) antioxidant activity, (**c**) sphericity factor (SF), and (**d**) roundness (Rn) values of the microcapsules, including the phenolic antioxidant-rich extract from rosemary (*Rosmarinus officinalis* L.).

Table 1 gives the design matrix of the ionic gelation process for the microencapsulation of the phenolic antioxidant-rich extract from rosemary (*Rosmarinus officinalis* L.) based on BBD–RSM. The selection of calcium chloride, sodium alginate, and time as process parameters was based on their critical impact on process efficiency and product quality, as reported in the literature [14,16,34,35] and preliminary experiments. Encapsulation efficiency (Y_1_) changed between 11.155% and 48.357% in terms of TPC. Free radical (DPPH) scavenging activity (Y_2_) was between 6.554 mg-TEAC/g-DM and 7.510 mg-TEAC/g-DM. While the sphericity factor (Y_3_) varied from 0.046 to 0.208, roundness (Y_4_) changed from 0.641 to 0.837. The analysis of variance (ANOVA) test is provided in Table 2. Figure 1, Figure 2, Figure 3 and Figure 4 represent the main effects of the process variables. Additionally, response surfaces (Figure 5, Figure 6 and Figure 7) were also produced by Design-Expert software (12.0.1.0 version, StatEase Inc., Minneapolis, MN, USA) in order to comprehend the effect of the process parameters on the selected responses.

#### 2.1.1. Impacts of Input Variables on the Encapsulation Efficiency

Figure 1 shows the main influences of the ionic gelation variables on the encapsulation efficiency in terms of total phenolic content. Figure 1a shows the effect of the calcium chloride concentration under an alginate concentration of 1.5% (*w*/*v*) and 20 min of hardening time to complete the gelation. Increasing the CaCl_2_ concentration enhances the efficiency at first. After the concentration reaches a certain value (>8.5% CaCl_2_), the yield declines as seen in Run 2, Run 4, Run 5, and Run 12, as seen in Table 1. Since the presence of calcium ions is required for the formation of gels in ionic gelation, the increase observed at the beginning is expected. Wani and Uppaluri reported a similar effect of CaCl_2_ on the encapsulation efficiency of papaya leaf microbeads in terms of TPC [17]. Essifi et al. also observed the same tendency for CaCl_2_ concentration’s impact on the EE of alginate beads loaded by gallic acid using the BBD–RSM approach [18]. Similarly, Liu et al. announced the same curvature on the response surfaces created by BBD–RSM during the ionic gelation of *Gardenia* yellow pigment-loaded alginate beads [15].

Figure 1b shows the alginate concentration effect under a CaCl_2_ concentration of 8.5% (*w*/*v*) and 20 min of hardening time to complete the gelation. Increasing the alginate concentration enhances the efficiency at first. There is an inversely proportional relationship between yield and alginate concentration (Run 2, Run 4, Run 5, and Run 12 in Table 1). Alginate is a key factor affecting the structure and pore size of the microbeads, as seen in Table 2 (*p* < 0.0001). As the amount of alginate increases, the gaps in the bead are filled with alginate, resulting in a structure with fewer voids. In this case, it reduces efficiency [35].

Hardening time, as the second most significant parameter after alginate concentration in the present process (*p* < 0.0001), is also inversely proportional to the EE, as seen in Figure 1c. Hardening time (time for gelation) is related to the trapping and dropping of the active material from the bead [36]. Increasing the gelation time from 10 min to 30 min decreased the EE yield by more than 26% under a CaCl_2_ concentration of 2% and an alginate concentration of 1.5%.

#### 2.1.2. Impacts of Input Variables on Antioxidant Activity

Figure 2 indicates the main effects of the ionic gelation variables on the scavenging activity of the produced microbeads against free radicals (DPPH). Figure 2a shows the effect of calcium chloride concentration on antioxidant activity under an alginate concentration of 1.5% (*w*/*v*) and 20 min of hardening time to complete the gelation. As seen in Run 2, Run 4, Run 5, and Run 12 in Table 1 and Figure 2a, the CaCl_2_ concentration in the covering solution decreases the antioxidant activity. It might be due to the release of the active substance with antioxidant properties due to the repulsion and attraction forces between Ca^++^ and Cl^-^ ions [15]. The antioxidant activity of the beads rises with the alginate concentration, as seen in Figure 2b. DPPH scavenging activity is enhanced by the alginate concentration since the release of the antioxidant active substance is prevented as the cover of the bead becomes thicker. In the case of the effect of time (Figure 2c), the increase in gelation time favors the antioxidant activity of the encapsulated products. An extended hardening time makes the gel harder and prevents the antioxidant substance from escaping [37].

#### 2.1.3. Impacts of Input Variables on Sphericity

Sphericity is a measure of the perfect shape for the controlled delivery of bioactive substances in applications [20]. The SF value is expected to be close to 0 for a perfect shape.

Figure 3 demonstrates the main influences of the ionic gelation variables on the sphericity of the produced particles. Figure 3a shows the effect of calcium chloride concentrations less than 1.5% (*w*/*v*) of the alginate concentration and 20 min of hardening time to complete the gelation. SF increases with CaCl_2_ concentrations, which is consistent with the observations of Jeong et al. [38]. SF is increased by increasing calcium lactate concentrations during the production of calcium alginate gels.

By increasing the alginate concentration, SF decreases, as seen in Figure 3b. Similarly, Gaikwad et al. reported the same effect of alginate concentration on the SF values of alginate beads loaded with pomegranate seed oil [39]. During the production of alginate beads loaded with black seed oil, the SF value also decreased with alginate levels [35]. This is ascribed to the viscous forces against the resistance in the gelling medium (calcium chloride solution). Hence, the more alginate there is, the more spherical beads are formed. Figure 3c shows the effect of hardening time under a calcium chloride concentration of 8.5% (*w*/*v*) and an alginate concentration of 1.5% (*w*/*v*). Time enhances the perfect shape, considering the drop in the SF value. When the time increased from 10 min to 30 min, SF decreased from 0.11 to 0.05 (2% CaCl_2_ and 1.5% alginate).

Roundness is another measurement for the representation of the spherical characteristics of perfect spheres. Rn is most desirable when it is close to 1, unlike SF. Figure 4a shows the effect of CaCl_2_ concentrations when the alginate concentration is 1.5% (*w*/*v*) and the hardening time to complete the gelation is 20 min. It has a very mild effect. An increase in CaCl_2_ enhances the Rn. As seen in Figure 4b, the alginate concentration also favors the roundness of the beads. Similarly, the Rn values of the alginate beads loaded with bioactive compounds extracted from the stems and leaves of *Beta vulgaris* were enhanced by calcium chloride concentrations, while the Rn increased with alginate concentrations [40]. Figure 4c indicates the effect of hardening time under a calcium chloride concentration of 8.5% (*w*/*v*) and an alginate concentration of 1.5% (*w*/*v*). Hardening time does not influence roundness, as reported in the ANOVA table (*p* > 0.5) in Table 3. Zazzali et al. also found that time had a non-significant effect on the Rn values of the alginate beads, including for artichoke waste extract [41].

#### 2.1.4. Modeling the Encapsulation Process

Design-Expert software (12.0.1.0) was used to apply a Box–Behnken design with three numeric factors to develop the final product with the maximum yields of encapsulation efficiency, antioxidant activity, and a perfect shape. Quadratic models were selected since the selected models maximized the adjusted R^2^ and the predicted R^2^. The following equations (Equations (1)–(4)) present the models in terms of coded factors:

(1) $$Y_{1}=33.23+2.35A-6.84B-6.32C+0.3348AB-2.95AC+2.43BC-8.61A^{2}-3.94\;B^{2}+3.64\;C^{2}$$

(2) $$Y_{2}=6.99-0.0691A+0.2080B+0.2479C-0.2424AB+0.1814AC-0.2022BC-0.0180A^{2}+0.253\;B^{2}+0.1986\;C^{2}$$

(3) $$Y_{3}=0.1250+0.0174A-0.0451B-0.0087C+0.0403AB+0.0215AC+0.0220BC+0.0011A^{2}+0.0166B^{2}-0.0241\;C^{2}$$

(4) $$Y_{4}=0.7616-0.0075A+0.0389B+0.0009C-0.0365AB-0.0470AC-0.0302BC+0.0026A^{2}-0.0527\;B^{2}+0.0098\;C^{2}$$

The ANOVA for the quadratic model is given in Table 2. Significant *p*-values (<0.0001), non-significant lack of fit values (>0.05), except for Y_2_, and R^2^ values (>0.98), including adjusted R^2^ (>0.97) and predicted R^2^ (>0.89), were all satisfying, showing that the quadratic model is suitable for making predictions of the four responses. Additionally, adjusted R^2^−predicted R^2^ < 0.2 demonstrates that both values are in reasonable agreement with each other, which is desired. On the other hand, the coefficient of variation (C.V.) < 6% indicates the reliability of the experimental data.

ANOVA also enables us to identify the influences of the numeric process factors on the responses (Table 2). First of all, alginate concentration (B) was the most effective parameter at *p* < 0.0001 for all of the responses except antioxidant activity. Actually, the effect of time (C) was more critical for the Y_2_, as it had the highest F-value (570.02). It was followed by alginate concentration, which had the second-highest F-value (401.37). For Y_1_, every term was found to be significant, but not the interaction between alginate and CaCl_2_ at *p* > 0.05. In the case of Y_2_, each term was effective significantly, except for the second power of CaCl_2_ (A^2^). Regarding SF, only A^2^ was non-significant, while C and A^2^ were not significantly effective at *p* > 0.05 for the roundness of the products.

Response surfaces were also drawn in order to observe the combined effects of the independent variables. Figure 5a–d indicate the influences of calcium chloride concentration (A) and sodium alginate concentration (B) on the encapsulation efficiency, antioxidant activity, sphericity factor, and roundness value of the microcapsules, including rosemary extract under 20 min of constant time (C), respectively. Figure 5a shows that increasing the CaCl_2_ concentration enhances the encapsulation efficiency at first. The yield declines after 8.5% of CaCl_2_. Alginate concentrations in the microbeads also have the same tendency towards EE. As seen in Figure 5b,d, antioxidant activity and Rn values are enhanced by alginate concentrations in the bead, regardless of CaCl_2_ content. While Rn increases (Figure 5d), SF expectedly decreases with alginate (Figure 5c).

Figure 6a–d demonstrate the influences of calcium chloride concentration (A) and hardening time (C) on the encapsulation efficiency, antioxidant activity, sphericity factor, and roundness value of the microcapsules, including rosemary extract under a constant sodium alginate concentration of 1.5% (*w*/*v*) (B), respectively. As seen in Figure 6a, EE is affected adversely by time regardless of the amount of calcium chloride concentration. Whatever the CaCl_2_ concentration, the DPPH scavenging activity (Figure 6b) and Rn value (Figure 6d) of the beads are favored by hardening time, whereas the SF value decreases. Figure 7a–d demonstrate the influences of alginate concentration (B) and hardening time (C) on the encapsulation efficiency, antioxidant activity, sphericity factor, and roundness value of the microcapsules, including rosemary extract under a constant CaCl_2_ concentration of 8.5% (*w*/*v*) (A), respectively. It is possible to see similar trends in the dependent factor values towards independent factors in these 3Ds as well.

**Figure 8:**
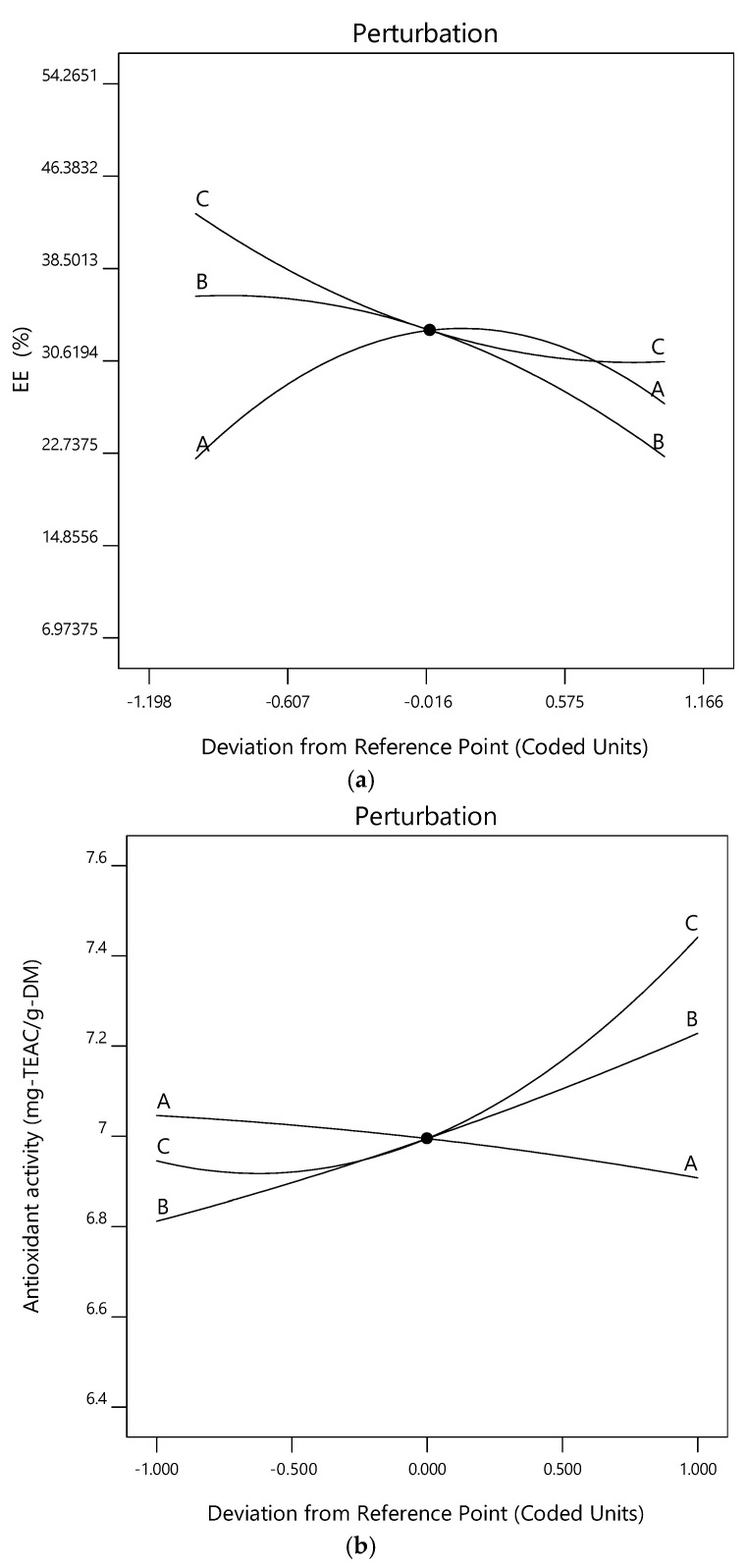

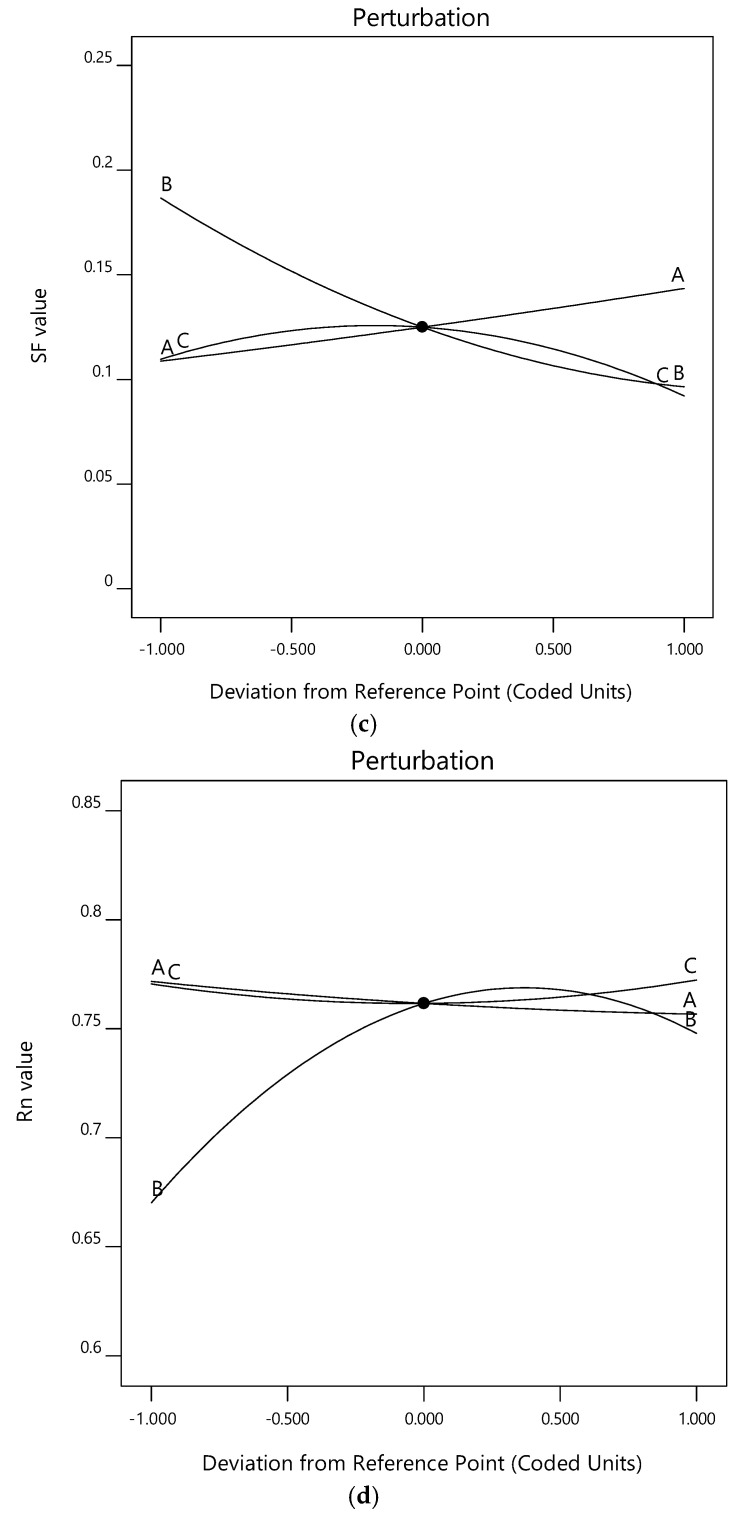
Perturbation plot for the (**a**) encapsulation efficiency, (**b**) antioxidant activity, (**c**) sphericity factor (SF), and (**d**) roundness (Rn) values of the microcapsules, including a phenolic antioxidant-rich extract from rosemary (*Rosmarinus officinalis* L.).

**Figure 9:**
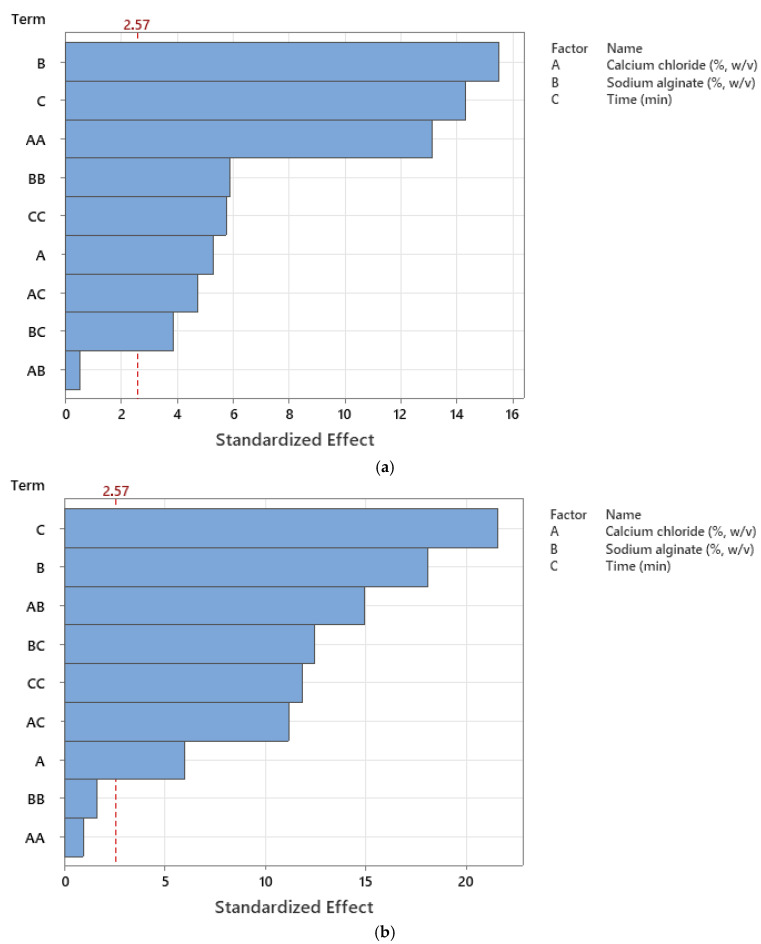

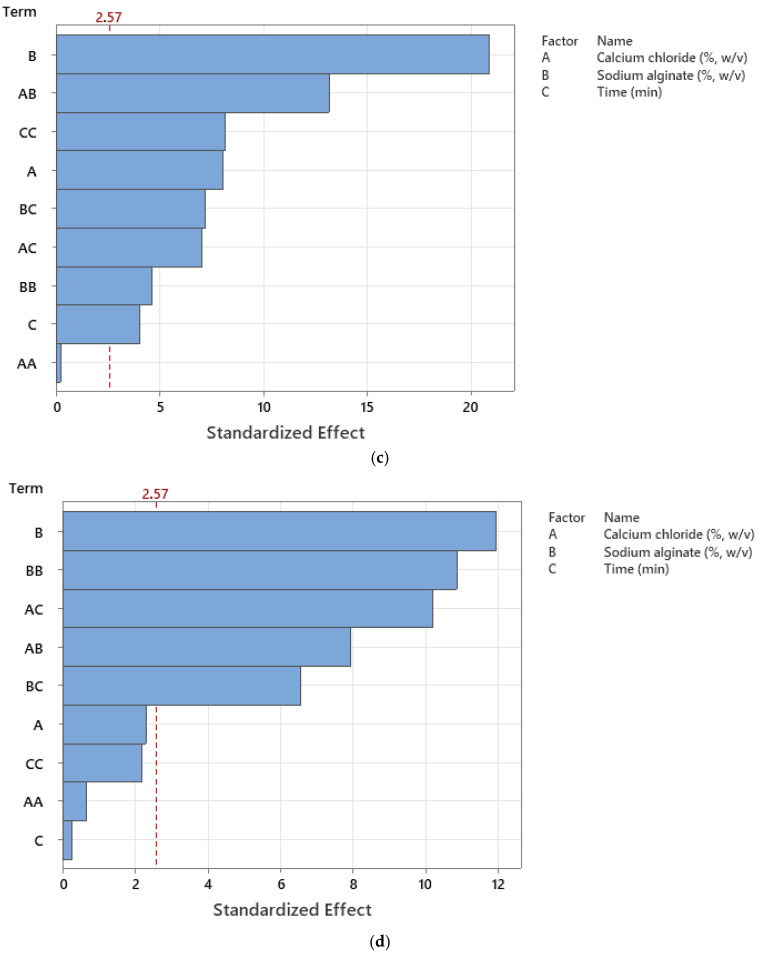
Pareto chart for the (**a**) encapsulation efficiency, (**b**) antioxidant activity, (**c**) sphericity factor (SF), and (**d**) roundness (Rn) values of the microcapsules, including a phenolic antioxidant-rich extract from rosemary (*Rosmarinus officinalis* L.).

Additionally, the perturbation plots for the models are given in Figure 8. The effects of the variables can also be seen in the related plots drawn using Design-Expert software (12.0.1.0 version). A steep slope or curvature in a factor shows that the response is sensitive to that factor. A relatively flat line shows insensitivity to change in that particular factor. On the other hand, Figure 9 also shows the effects of each term on the relevant responses through Pareto charts produced using Minitab software (22 version), in addition to Figure 1, Figure 2, Figure 3 and Figure 4.

#### 2.1.5. Verification of the Estimated Values

The limitations and the optimum solutions for the best formulation of microbeads, including the phenolic antioxidant-rich rosemary extract, are given in Table 3. Weight and importance are considered to be equal for the four responses (EE, antioxidant activity, SF, and Rn). Under the given constraints, the optimum conditions are calculated as ~6% CaCl_2_, ~2% sodium alginate, and 10 min hardening time for the best formulation of microbeads. The differences between the actual and estimated findings were satisfactory (less than 2%) under the given conditions based on the validation study.

### 2.2. Characterization Analysis of the Microbeads

After the optimal conditions were identified, the verification study was performed as stated above. The products produced under their optimal points were subjected to quality measurements, such as shape and size, and some physicochemical properties (moisture content, water activity, and bulk density).

#### 2.2.1. Shape and Size

The imaging analyses of microbeads produced under optimal conditions measured their d_min_, d_max_, and d_average_ values. Based on these results, SF and Rn values were calculated. The d_max_, d_min_, and d_average_ values of the microcapsules were measured as 142 ± 1.15 µm, 125 ± 3.96 µm, and 133 ± 1.98 µm, respectively. When the microcapsule images were analyzed, the SF value was measured as 0.04 ± 0.02 and the Rn value as 0.79 ± 0.01. The calculated results indicate that they are acceptable and very close to sphericity. The studies specify that an SF value below 0.05 is desired [42]. The drying method also plays a role in determining the final shape of microcapsules produced through ionic gelation. Different drying methods directly impact the shape of microcapsules obtained through ionic gelation. Additionally, the diameter, SF, and Rn values of hydrogel beads exhibited significant variability across different studies due to varying process conditions, such as gelation temperature, hardening time, and concentrations of sodium alginate and calcium chloride [37].

#### 2.2.2. Moisture Content, Water Activity, and Bulk Density

The moisture content, water activity, and bulk density of microcapsules were 75.58 ± 1.48%, 0.48 ± 0.01, and 0.34 ± 0.01 g/mL, respectively. In general, the high water-absorbing capacity of alginate results in the formation of a 3D network with a high moisture content. However, the drying process of the capsules after treatment directly affects the moisture content. Conversely, the moisture content and density of the hydrogels are influenced by the nature of the core material. An oily core tends to decrease the moisture content and density [37,43]. Similarly, our findings are in agreement with those of Najafi-Soulari et al. on the encapsulation of lemon balm antioxidants in alginate gels [11]. The moisture content, water activity, and bulk density values were reported as 97.81 ± 0.37%, 0.98 ± 0.00, and 0.76 ± 0.01 g/cm^3^, respectively.

## 3. Conclusions

Microbeads were produced by trapping aqueous alcoholic rosemary extract in alginate through the ionic gelation method. The statistical findings of the ANOVA test for modeling produced satisfying *p*-values (at *p* < 0.0001), non-significant lack of fit values (*p* > 0.05), R^2^ values (>0.98), including adjusted R^2^ (>0.97) and predicted R^2^ (>0.89), and C.V. < 6% for estimations of EE, antioxidant activity, SF, and Rn responses. Additionally, the optimized conditions for the best formation of the microbeads were achieved with a ~6% calcium chloride concentration, a 2% alginate concentration, and a 10 min hardening time. The information obtained from this study is important preliminary data as it involves the entrapment of the active substance in alginate for the first time. In future studies, release/digestion studies can be carried out to investigate the release kinetics and bioavailability of encapsulated phenolic compounds.

## 4. Materials and Methods

### 4.1. Materials

The dried rosemary (*Rosmarinus officinalis* L.) samples cultivated in Turkey were purchased from a commercial organization. The samples were ground and classified according to particle size. Among the particle sizes of 0.5, 1, and 1.5 mm, the highest efficiency was achieved with 1.5 mm. Therefore, particles of this size were used in the extraction study. It was determined that a particle size of 1.5 mm provided the highest efficiency since these results were supported by total phenolic content (TPC) analysis.

All chemical materials (Folin–Ciocalteu reagent, sodium carbonate, 2,2-diphenyl-1-picrylhydrazil (DPPH), ethanol, methanol, 6-hydroxy-2,5,7,8-tetramethylchroman-2-carboxylic acid (trolox), gallic acid monohydrate, sodium alginate, and calcium chloride dihydrate) were provided by Sigma-Aldrich (St. Louis, MO, USA). The purity of the chemicals is as follows: ethanol (≥99.8%), methanol (≥99.9%), DPPH (≥95%), trolox (≥98%), gallic acid monohydrate (≥99%), sodium carbonate (≥99.5%), sodium alginate (≥98%), and calcium chloride dihydrate (≥96%). Our commercial sodium alginate has a viscosity of 20–400 cP (1% aqueous solutions) [44]. Zahoor et al. also identified a similar brand of sodium alginate as being of medium viscosity (3500 cP for a 2% *w*/*v* solution) [45]. Its molecular weight is 216.121 g/mol.

### 4.2. Extraction of Phenolic Antioxidants from Rosemary

An automatic solvent extractor (Velp Scientifica, Usmate, Italy) was used in this step of the study. This method was particularly preferred since the current study aimed for a green process. The solvent and time consumption of this technology are low compared to conventional extraction methodologies. Additionally, it works with extremely high efficiency [46]. Speaking of green technology, an aqueous solution of ethanol, which is also certified as GRAS (generally recognized as safe) by the United States Food and Drug Administration, was used as the solvent. Since pure ethanol was insufficient for the extraction of phenolic components, 20% water was added, owing to its swelling effect on the plant matrix, increasing the diffusivity of the target substance. Consequently, 80 mL of solvent (80% ethanol) was introduced into 0.5 g of solid. Table 4 presents the process conditions applied during the extraction of phenolic antioxidants from rosemary.

### 4.3. Encapsulation of Rosemary Phenolic Antioxidants in Alginate Microbeads

The ionic gelation method, in the presence of a calcium chloride solution (as a gelling medium), was used to produce alginate microbeads loaded with rosemary extract. Table 5 provides the selected variables. The conditions were designed and optimized using a statistical experimental design (BBD–RSM) in order to create the best formulation with the highest quality. The rosemary extract/alginate solution was maintained constantly at a ratio of 1/2 (*v*/*v*). The alginate solution and extract were mixed homogeneously. The physical state of the system can be described as a viscous solution. This was formed as a result of the dissolution of alginate in water and its interaction with the extract.

A total of 30 mL of the rosemary extract/alginate solution was introduced into the 250 mL of calcium chloride solution over a magnetic stirrer (Model MSH-20D, DAIHAN Scientific Co. Ltd., Wonju, Republic of Korea). The stirring conditions were adjusted to 270 rotations per minute at room temperature. A syringe tip (pipette tip) that provides droplet formation with a constant diameter was used for this process. After the microbeads were generated, the products were kept for a while (10 min, 20 min, and 30 min) and examined as parameters. Later, the products were filtered and washed with water. The cleaned microbeads were dried before quality analysis.

### 4.4. Physicochemical Measurements

The water activity (aw) value of the samples was determined at room temperature (25 ± 1 °C) using a water activity determination device (AquaLab, 4TE, Pullman, WA, USA). The moisture content of the capsules was determined by drying the samples in an oven (70 °C and 24 h). Additionally, the bulk density (ρ_b_) of the samples was calculated from the mass/volume ratio [37].

### 4.5. Measurement of Sphericity and Roundness

After 17 microbeads were produced depending on the ionic gelation conditions, they were analyzed with a stereo-microscope (Stemi 2000-C, Zeiss, Göttingen, Germany) and an AxioCamERc5 camera (Stemi 2000-C, Zeiss, Göttingen, Germany). In order to determine the sphericity factor (SF), d_max_ (maximum diameter), d_min_ (minimum diameter), P (perimeter), and A (area) values of the capsules, they were measured with ZEN lite, 2012 software. SF was calculated using Equation (5) [42]:

(5) $$Spherical\;factor\;(SF)=\frac{d_{max}-d_{min}}{d_{max}+d_{min}}$$

Equation (6) gives the roundness (Rn) values of the beads, calculated by the parameters measured by the same software:

(6) $$Roundness\;(Rn)=\frac{P^{2}}{4\pi A}$$

### 4.6. Encapsulation Efficiency

Encapsulation efficiency (EE) is a significant measurement for the quantification of the success of the encapsulation system. In this study, EE was calculated with respect to TPC [47]. Equation (7) was used to evaluate the EE value as a percentage:

(7) $$EE\left(\%\right)=\frac{TPC-SPC}{TPC}\times 100$$

The TPC results measured at a wavelength of 760 nm were presented as the mg gallic acid equivalent (GAE)/g dried microbead (DM). A total of 100 mg of microcapsules in 3 mL of an ethanol–acetic acid–water mixture (50/8/42, *v*/*v*/*v*) was mixed through a vortex for 1 min. Then, ultrasound treatment was applied to the mixture at ambient conditions. The mixture (100 µL) was put into a 10% Folin–Ciocalteau reagent (2000 µL) and incubated for 5 min. Later, 7.5% sodium carbonate solution (1800 µL) was introduced into the final mixture, which was left in the dark for 1 h.

The surface phenolic content (SPC) of the products was also calculated in order to express the EE results. A 100 mg microbead was dissolved in ethanol–methanol solution (1/1, *v*/*v*) for 5 min by means of an ultrasonic bath (Protech, Istanbul, Turkey) under ambient conditions [48]. This is also given in mg-GAE/g-DM.

### 4.7. DPPH Scavenging Activity

The antioxidant activity of the produced beads was measured using the 2,2-diphenyl-1-picryl-hydrazyl-hydrate (DPPH) method at 517 nm. Briefly, a 15 mg sample was dissolved in an ethanol/acetic acid/water (50:8:42, *v*:*v*:*v*) solution. After vortexing (1 min) and water bath (40 °C for 20 min) treatments, DPPH solution was introduced into the sample solution. Then, the mixture was left for 1 h in the dark. The calibration curve was drawn using the trolox standard. The results were given in trolox equivalents (TEAC) per g dried microbead.

### 4.8. BBD–RSM

The study design consists of 3 numeric process factors, as given in Table 5. Design-Expert software (12.0.1.0 version, StatEase Inc., Minneapolis, MN, USA) was used. The Box–Behnken design produced 17 randomized experimental runs. On the other hand, the effects of process parameters on the ionic gelation process and model fitting were determined by analysis of variance (ANOVA) tests using the software (Design-Expert). F-values, P-values, lack-of-fit values, coefficient of variance (C.V.), coefficient of variation (R^2^), and adjusted R^2^ and predicted R^2^ were used for statistical analysis. Additionally, Minitab statistical software 22 (Minitab Inc., State College, PA, USA) was also utilized for drawing the Pareto charts.

## Figures and Tables

**Table 1:** Experimental results formed by BBD–RSM for the microencapsulation of the phenolic antioxidant-rich extract from rosemary (*Rosmarinus officinalis* L.) *.

Run	A (%, *w*/*v*)	B (%, *w*/*v*)	C (min)	Y_1_ (%)	Y_2_ (mg-TEAC/g-DM)	Y_3_	Y_4_
1	2	1.5	30	22.388	7.316 ± 0.003	0.052 ± 0.02	0.837 ± 0.01
2	8.5	1.5	20	33.344	7.018 ± 0.000	0.113 ± 0.02	0.762 ± 0.01
3	8.5	2	10	29.085	7.359 ± 0.002	0.054 ± 0.02	0.792 ± 0.01
4	8.5	1.5	20	31.616	6.983 ± 0.001	0.131 ± 0.04	0.759 ± 0.03
5	8.5	1.5	20	34.183	7.000 ± 0.001	0.127 ± 0.04	0.770 ± 0.02
6	2	1.5	10	30.172	7.212 ± 0.000	0.114 ± 0.03	0.731± 0.02
7	8.5	2	30	22.368	7.479 ± 0.006	0.082 ± 0.01	0.723 ± 0.02
8	2	2	20	11.155	7.510 ± 0.000	0.046 ± 0.02	0.792 ± 0.02
9	15	2	20	17.255	6.926 ± 0.000	0.158 ± 0.03	0.709 ± 0.02
10	8.5	1	10	48.357	6.554 ± 0.002	0.197 ± 0.04	0.654 ± 0.03
11	15	1.5	30	20.438	7.502 ± 0.001	0.133 ± 0.02	0.723 ± 0.02
12	8.5	1.5	20	33.288	6.989 ± 0.001	0.128 ± 0.02	0.767 ± 0.02
13	15	1	20	29.536	6.979 ± 0.001	0.159 ± 0.03	0.704 ± 0.05
14	8.5	1.5	20	33.730	6.983 ± 0.001	0.126 ± 0.03	0.765 ± 0.01
15	8.5	1	30	31.929	7.483 ± 0.004	0.137 ± 0.05	0.706 ± 0.03
16	2	1	20	24.775	6.593 ± 0.000	0.208 ± 0.06	0.641 ± 0.05
17	15	1.5	10	40.035	6.672 ± 0.003	0.109 ± 0.03	0.805 ± 0.03

* Data are given as the arithmetic mean of 3 replicates.

**Table 2:** ANOVA for the quadratic models.

**Y_1_**	**Source**	SS *	DF **	MS ***	**F-Value**	***p*-Value**	
	Model	1227.10	9	136.34	116.65	<0.0001	Significant ****
	A	44.06	1	44.06	37.70	0.0005	
	B	374.46	1	374.46	320.36	<0.0001	
	C	319.12	1	319.12	273.02	<0.0001	
	AB	0.4483	1	0.4483	0.3836	0.5553	
	AC	34.88	1	34.88	29.84	0.0009	
	BC	23.58	1	23.58	20.17	0.0028	
	A^2^	312.44	1	312.44	267.30	<0.0001	
	B^2^	65.29	1	65.29	55.86	0.0001	
	C^2^	55.80	1	55.80	47.74	0.0002	
	Residual	8.18	7	1.17			
	Lack of Fit	4.40	3	1.47	1.55	0.3317	not significant
	Pure Error	3.78	4	0.9446			
	Cor Total	1235.28	16				
	C.V.: 3.72% R^2^ = 0.9934 Adjusted R^2^ = 0.9849 Predicted R^2^ = 0.9382						
	**Source**	**SS**	**DF**	**MS**	**F-Value**	***p*-Value**	
**Y_2_**	Model	1.58	9	0.1753	203.27	<0.0001	significant
	A	0.0382	1	0.0382	44.30	0.0003	
	B	0.3461	1	0.3461	401.37	<0.0001	
	C	0.4915	1	0.4915	570.02	<0.0001	
	AB	0.2351	1	0.2351	272.59	<0.0001	
	AC	0.1316	1	0.1316	152.64	<0.0001	
	BC	0.1635	1	0.1635	189.57	<0.0001	
	A^2^	0.0014	1	0.0014	1.58	0.2490	
	B^2^	0.0027	1	0.0027	3.12	0.1204	
	C^2^	0.1660	1	0.1660	192.56	<0.0001	
	Residual	0.0060	7	0.0009			
	Lack of Fit	0.0052	3	0.0017	7.82	0.0378	significant
	Pure Error	0.0009	4	0.0002			
	Cor Total	1.58	16				
	C.V.: 0.4141% R^2^ = 0.9962 Adjusted R^2^ = 0.9913 Predicted R^2^ = 0.9470						
	**Source**	**SS**	**DF**	**MS**	**F-Value**	***p*-Value**	
**Y_3_**	Model	0.0330	9	0.0037	68.92	<0.0001	significant
	A	0.0024	1	0.0024	45.35	0.0003	
	B	0.0163	1	0.0163	305.92	<0.0001	
	C	0.0006	1	0.0006	11.50	0.0116	
	AB	0.0065	1	0.0065	121.69	<0.0001	
	AC	0.0018	1	0.0018	34.72	0.0006	
	BC	0.0019	1	0.0019	36.36	0.0005	
	A^2^	5.329 × 10^−6^	1	5.329 × 10^−6^	0.1001	0.7610	
	B^2^	0.0012	1	0.0012	21.85	0.0023	
	C^2^	0.0025	1	0.0025	46.02	0.0003	
	Residual	0.0004	7	0.0001			
	Lack of Fit	0.0002	3	0.0001	1.23	0.4087	not significant
	Pure Error	0.0002	4	0.0000			
	Cor Total	0.0334	16				
	C.V.: 5.98% R^2^ = 0.9888 Adjusted R^2^ = 0.9745 Predicted R^2^ = 0.9053						
	**Source**	**SS**	**Df**	**MS**	**F-Value**	***p*-Value**	
**Y_4_**	Model	0.0423	9	0.0047	74.46	<0.0001	significant
	A	0.0004	1	0.0004	7.14	0.0319	
	B	0.0121	1	0.0121	191.71	<0.0001	
	C	6.125 × 10^−6^	1	6.125 × 10^−6^	0.0971	0.7644	
	AB	0.0053	1	0.0053	84.50	<0.0001	
	AC	0.0088	1	0.0088	140.11	<0.0001	
	BC	0.0037	1	0.0037	58.04	0.0001	
	A^2^	0.0000	1	0.0000	0.4427	0.5271	
	B^2^	0.0117	1	0.0117	185.25	<0.0001	
	C^2^	0.0004	1	0.0004	6.44	0.0387	
	Residual	0.0004	7	0.0001			
	Lack of Fit	0.0003	3	0.0001	1.92	0.2687	not significant
	Pure Error	0.0002	4	0.0000			
	Cor Total	0.0427	16				
	C.V.: 1.07% R^2^ = 0.9897 Adjusted R^2^ = 0.9764 Predicted R^2^ = 0.8959						

* SS: Sum of squares. ** DF: Degrees of freedom. *** MS: Mean square. **** Statistically significant (<0.05) or non-significant (>0.05).

**Table 3:** The constraints and solutions for optimal ionic gelation conditions for the microencapsulation of a phenolic antioxidant-rich extract from rosemary (*Rosmarinus officinalis* L.).

Criteria						
Name	Goal	Lower Limit	Upper Limit	Lower Weight	Upper Weight	Importance
A	is in range	2	15	1	1	3
B	is in range	1	2	1	1	3
C	is in range	10	30	1	1	3
EE	Maximize	11.155	48.357	1	1	3
Antioxidant activity	Maximize	6.554	7.510	1	1	3
SF	Minimize	0.046	0.208	1	1	3
Rn	Maximize	0.641	0.837	1	1	3
**Solutions**						
**A (%, *w*/*v*)**	**B (%, *w*/*v*)**	**C (min)**	**Response**	**Predicted**	**Experimental**	**Error (%)**
6	2	10	EE	29.48	30.01	<2
			(%)			
			Antioxidant activity (mg-TEAC/g-DM)	7.47	7.55	
			SF	0.05	0.05	
			Rn	0.79	0.78	

**Table 4:** Extraction procedure of the phenolic antioxidant-rich extract from rosemary (*Rosmarinus officinalis* L.).

Process	Processing Time (min)
Immersion	30
Washing	45
Recovery	20
Cooling	5

**Table 5:** Input and output of ionic gelation for the microencapsulation of the phenolic antioxidant-rich extract from rosemary (*Rosmarinus officinalis* L.).

Independent Variable	Coded Levels		
	−1	0	1
A: Calcium chloride (%, *w*/*v*)	2	8.5	15
B: Sodium alginate (%, *w*/*v*)	1	1.5	2
C: Time (min)	10	20	30
Dependent variable *			
Y_1_: EE (%)			
Y_2_: Antioxidant activity (mg-TEAC/g-DM)			
Y_3_: SF			
Y_4_: Rn			

* EE: Encapsulation efficiency, SF: Sphericity factor, Rn: Roundness.

## Data Availability

The datasets generated during and/or analyzed during the current study are available from the corresponding author on reasonable request.
